# Endothelial Nitric Oxide Synthase Is Present in Dendritic Spines of Neurons in Primary Cultures

**DOI:** 10.3389/fncel.2017.00180

**Published:** 2017-07-04

**Authors:** Ariel Caviedes, Manuel Varas-Godoy, Carlos Lafourcade, Soledad Sandoval, Javiera Bravo-Alegria, Thilo Kaehne, Angela Massmann, Jorge P. Figueroa, Francisco Nualart, Ursula Wyneken

**Affiliations:** ^1^Laboratorio de Neurociencias, Centro de Investigación Biomédica, Facultad de Medicina, Universidad de los AndesSantiago, Chile; ^2^Laboratorio Biología de la Reproducción, Centro de Investigación Biomédica, Facultad de Medicina, Universidad de los AndesSantiago, Chile; ^3^Institute of Experimental Internal Medicine, Otto-von-Guericke UniversityMagdeburg, Germany; ^4^Department of Obstetrics and Gynecology, Wake Forest School of Medicine, Winston-SalemNC, United States; ^5^Centro de Microscopía Avanzada, CMA BIO BIO, Laboratorio de Neurobiología y Células Madres, Facultad de Ciencias Biológicas, Universidad de ConcepciónConcepción, Chile

**Keywords:** eNOS, nitric oxide, dendritic spines, excitatory synapses

## Abstract

Nitric oxide exerts important regulatory functions in various brain processes. Its synthesis in neurons has been most commonly ascribed to the neuronal nitric oxide synthase (nNOS) isoform. However, the endothelial isoform (eNOS), which is significantly associated with caveolae in different cell types, has been implicated in synaptic plasticity and is enriched in the dendrites of CA1 hippocampal neurons. Using high resolution microscopy and co-distribution analysis of eNOS with synaptic and raft proteins, we now show for the first time in primary cortical and hippocampal neuronal cultures, virtually devoid of endothelial cells, that eNOS is present in neurons and is localized in dendritic spines. Moreover, eNOS is present in a postsynaptic density-enriched biochemical fraction isolated from these neuronal cultures. In addition, qPCR analysis reveals that both the nNOS as well as the eNOS transcripts are present in neuronal cultures. Moreover, eNOS inhibition in cortical cells has a negative impact on cell survival after excitotoxic stimulation with *N*-methyl-D-aspartate (NMDA). Consistent with previous results that indicated nitric oxide production in response to the neurotrophin BDNF, we could detect eNOS in immunoprecipitates of the BDNF receptor TrkB while nNOS could not be detected. Taken together, our results show that eNOS is located at excitatory synapses where it could represent a source for NO production and thus, the contribution of eNOS-derived nitric oxide to the regulation of neuronal survival and function deserves further investigations.

## Introduction

Nitric oxide (NO) is a free radical gas with pivotal signaling capacities in the central nervous system (Benarroch, 2011; Hardingham et al., 2013). It has a recognized role in the regulation of synaptic plasticity, excitability and excitotoxicity (Calabrese et al., 2007; Brunert et al., 2009; Steinert et al., 2010, 2011).

The principal cellular source of NO is its synthesis by three nitric oxide synthase (NOS) isoforms that were cloned in neurons, immune cells and endothelial cells, respectively, thus receiving their characteristic denominations, namely, neuronal NOS (nNOS, type I); inducible NOS (iNOS, type II) and endothelial NOS (eNOS, type III) (Guix et al., 2005). However, it is now accepted that the cell and tissue distribution of these isoforms is much wider than previously suspected (Lowenstein and Michel, 2006). In addition, the expression levels of nNOS and eNOS, originally thought to be constitutive isoforms, fluctuate in response to different stimuli while their activity is tightly regulated by Ca^2+^-calmodulin and several post-translational modifications (Qian and Fulton, 2013; Heiss and Dirsch, 2014). Although nNOS has been historically associated with the actions of NO in the central nervous system (CNS), the expression pattern of this isoform is restricted to inhibitory interneurons and to a small population of excitatory neurons in the cerebral cortex and hippocampus (Wendland et al., 1994; Jinno and Kosaka, 2002), in which the enzyme seems to be confined to dendritic spines (Aoki et al., 1998). In contrast, eNOS which is known for its prominent role in the regulation of cerebral blood flow, was surprisingly detected in neocortical and hippocampal neurons where it constitutes a primary source of NO necessary for the induction of long-term potentiation (LTP), while membrane targeting is necessary to induce plasticity (Dinerman et al., 1994; O’Dell et al., 1994; Roskams et al., 1994; Haley et al., 1996; Kantor et al., 1996; Haul et al., 1999). Notwithstanding, the putative localization of eNOS in the brain has been highly controversial: while some authors have found it exclusively in blood vessels, others find a punctate staining in human pyramidal hippocampal neurons (Doyle and Slater, 1997; Blackshaw et al., 2003). eNOS is commonly associated to plasma membrane caveolae or intracellularly, to the Golgi apparatus through two major lipid modifications: myristoylation and/or palmitoylation at two cysteine residues (Brosnihan et al., 2008; Heiss et al., 2015). Surprisingly, the potential localization of eNOS to synaptic sites has not been further addressed. We therefore used primary neuronal cultures, a system free of endothelial cells, to assess eNOS localization in hippocampal and cortical neurons by confocal and super-resolution microscopy as well as by Western Blot and qPCR.

## Materials and Methods

### Materials

Chemical reagents were purchased from Sigma (St. Louis, MO, United States), unless otherwise stated. Neurobasal medium (Cat. N°: 21103-049), B27 (Cat. N° 17504-044), FBS (Fetal Bovien Serum) (Cat. N° 10438026) and MEM (Minimum Essential Medium Cell Culture, Cat. N° 11900-024) were from Gibco-Invitrogen (San Diego, CA, United States). Equine Serum (Cat. N° SH30074.03) were from HyQ Hyclone (Logan, UT, United States). Penicillin-Streptomycin-Amphotericin B Solution (Cat. N 03-033-1B) was from Biological Industries (Andes Import, Chile). The following primary antibodies were used (Supplementary Table S1): Anti-eNOS (BD Transduction laboratories, Cat. N° 610297), Anti-phospho-eNOS (BD Transduction Laboratories, Cat. N° 612393), Anti-nNOS (BD Transduction Laboratories, Cat. N° 610308), Anti-PSD-95 (BD Transduction Laboratories, Cat. N° 610495), Anti-MAP2 (Millipore, Cat. N° AB5622), Anti-synapthophysin (Abcam, Cat. N° ab14692), Anti-Thy1 (Abcam, Cat. N° ab92574), Anti-caveolin1 (Abcam, Cat. N° ab2910), and Anti-SHANK3/ProSAP2 (gift of Dr. Eckart Gundelfinger, Magdeburg, Germany) as used in Haeckel et al. (2008). Anti-eNOS (Abcam, Cat. N° Ab66127) was used in **Supplementary Figure S3**. *Secondary antibodies*: Alexa Fluor^®^ 488 Donkey Anti-Mouse IgG (Cat. N° A21202) and Alexa Fluor^®^ 555 Donkey Anti-Rabbit IgG (Cat. N° A31570) were from Invitrogen Corporation, (Molecular Probes, EEUU). Alexa Fluor^®^ 488 Goat Anti-Rabbit IgG (Cat. N° A11034) and Alexa Fluor^®^ 555 Goat Anti-Rabbit IgG (Cat. N° A21429), were from Life Technologies.

### Animals

This study was carried out in accordance with the recommendations of the National Institute of Health Guide for the Care and Use of Laboratory Animals. The protocol was approved by the Universidad de los Andes Animal Care and Use Committee in the frame of the Fondecyt Project 1140108.

### Neuronal Cultures

Primary cultures of cortical and hippocampal neurons were obtained from rat embryos (day 18) as previously described to perform immunofluorescent stainings or to obtain a Triton-insoluble cellular fraction (Sandoval et al., 2011). As described, these cultures contained less than 20% of glial cells, staining positively for glial fibrillary acid protein (GFAP, not shown) that were virtually not detectable when the cells were grown in the presence of an inhibitor of glial cell proliferation, 2 μM Cytosine β-D-arabinofuranoside (AraC) (Sigma C1768), added 24 h after plating (Schwieger et al., 2016).

### Astrocyte Cultures

Astrocyte cell cultures were performed from rat cerebral cortices of foetuses of 21 days of gestation. Cells were mantained in DMEM/F12 Ham (Sigma) containing 10% FBS with 100 units/ml of penicillin and 100 μg/ml of streptomycin. The medium was changed twice a week. After 15 days in culture, microglial cells were discarded by shaking the flask while astrocytes were further purified by trypsinization of the attached cells to re-plate them at low density and allow proliferation to reach 70–90% confluence for RNA extraction.

### Design and Subcloning of Short Hairpin RNA

Inverted and self-complementary DNA oligos targeting Rattus norvegicus endothelial nitric oxide synthase (eNOS) mRNA were chemically synthesized (IDT), aligned and ligated between the HpaI and XhoI sites (downstream the U6 promoter) of the lentiviral vector pLL3.7-mRuby2 containing a CMV-driven RFP reporter mRuby2 (Brummelkamp et al., 2002;Rubinson et al., 2003). The sequence for the Rattus norvegicus eNOS shRNAs (sh-eNOS) was: 5′-GTGTGAAGGCGACTATCCTGTATGGCTCT-3′ (shRNA1) or 5′-CACAGACGGAAGATGTTCCAGGCTACAAT-3′ (shRNA2) The scrambled sequence was: 5′-GGTAGAGTTGTTATGTGTAA-3′. Correct insertions of shRNA cassettes were confirmed by restriction mapping and direct DNA sequencing.

### Lentivirus Production

Lentiviral production was done using the calcium phosphate method. Briefly, we co-transfected the sh-eNOS or sh-scrambled plasmids with the packaging vector Δ8.91 and the envelope vector VSV-g into HEK293T cells, and the medium was replaced 16 hours after transfection for neurobasal serum free medium (Gibco). The resulting supernatant containing the lentiviruses was harvested after 60 hours, centrifuged to eliminate cell debris, and filtered through 0.45-mm cellulose acetate filters (Naldini et al., 1996; Dull et al., 1998).

### Quantitative RT-PCR

For gene expression profile, total RNA from primary cultures of cortical (CX), hippocampal (HP) neurons, astrocyte (AST), and mixed neuron/glia culture was extracted using TRIzol reagent (Life Technologies). For knockdown experiments, neuronal cultures of 3 days *in vitro* (DIV) were transduced with lentiviral vector encoding a scrambled shRNA or a shRNA against eNOS, with a 40–50% transduction efficiency in at least 4 independent neuronal cultures. Total RNA was extracted 4 days after transduction using TRIzol reagent (Life Technologies). 1 μg of RNA was reverse transcribed into cDNA using MultiScribe reverse transcriptase (ThermoFisher) according to the manufacturer’s protocol. Quantitative polymerase chain reaction (qPCR) reaction was carried out using the Brilliant III Ultra Fast QPCR Master Mix (Agilent Technologies, United States) in the Stratagene Mx3000P system (Agilent Technologies, Santa Clara, United States). The thermal cycling protocol was: pre-incubation, 95°C, 10 min; amplification, 40 cycles of (95°C, 20 s; 60°C, 20 s; 72°C, 20 s); melting curve, 1 cycle of (95°C, 1 min; 55°C, 30 s; 95°C, 30 s). qPCR was performed using duplicates. Primers used were: rat eNOS, forward primer 5′ ATTCTGGCAAGACCGATTAC 3′ and reverse primer 5′ TAGAGATGGTCCAGTTGGG 3′, rat nNOS, forward primer 5′ GGAACCCTTGCGTTTCTT 3′ and reverse primer 5′ CTGTTGAATCGGACCTTGTAG 3′. The results were normalized against rat mRNA of beta actin (forward primer 5′ CACAGCTGAGAGGGAAATC 3′; reverse primer 5′ TCAGCAATGCCTGGGTAC 3′). The threshold cycle (Ct) of each sample was determined, and the gene expression was represented by the ΔCt value (test Ct – housekeeping Ct). The relative expression was expressed as a fold change using arbitrary units.

### Immunofluorescence

Neuronal cultures of 18–21 DIV were fixed in 4% paraformaldehyde in phosphate buffered saline (PBS) containing 4% of sucrose for 10 min and washed with PBS. After fixation, the cells were permeabilizated with 0.2% triton X-100 for 5 min and washed with PBS containing 25 mM glycine. Cells were incubated with blocking solution [10% bovine serum albumin (BSA) in PBS] for 1 h followed by overnight incubation with primary antibody diluted in the same blocking solution at 4°C. After incubation with primary antibody, cells were washed with PBS, blocked for 30 min with 10% BSA and incubated for 1 hour with the corresponding secondary antibody and analyzed using confocal laser microscopy (Carl Zeiss, LSM700, Axio Observer.Z1) and structural illumination microscopy (SIM)/super resolution (SR) laser microscopy (Carl Zeiss, Elyra S1 SR-SIM, Axio Observer.Z1 HR). Confocal images provide a lateral resolution of around 300 nm, while dendritic spines have an average width of 600 nm (Carmona et al., 2009). However, the pre-synaptic bouton is in close contact to the postsynaptic density containing the scaffolding proteins we used to label excitatory synapses at the post-synaptic level. Thus, the use of SR-SIM is helpful to indicate a preferential pre- vs. post-synaptic distribution of a protein as lateral resolution is improved to ∼100 nm (Gustafsson et al., 2008; Schermelleh et al., 2010). Images were collected from *n* = 4 to 7 independent cell cultures, performed on different dates to analyze 6 to 8 neurons per culture dish. Image analysis was done with the IMARIS 6.0 software, confocal images were deconvoluted with Autoquant X2 software and correlation coefficients calculated according to Mander’s (Dunn et al., 2011).

### Excitotoxicity Assay

The excitotoxic assay consisted in exposure of neurons to 30 μM NMDA for 1 h, as described previously by us. In addition, 10 μM 6-cyano-7-nitroquinoxaline-2,3-dione (CNQX), 2 μM nimodipine, and 1 μM tetrodotoxin (TTX) were added to block α-amino-3-hydroxy-5-methyl-4-isoxazolepropionic acid (AMPA) receptor, Ca2+ and Na^+^ channels. This was done in the presence of 1 μM of 7-Nitroindazole (7-NI), a preferential nNOS inhibitor or 10 μM of N5-(1-Iminoethyl)-L-ornithine (LNIO), a preferential eNOS inhibitor. The cell death was assessed 24 h later with the trypan blue exclusion test by incubation with 0.05% (v/v) trypan blue in PBS for 5 min. Stained neurons (i.e., death neurons) were quantified in random images taken with a phase-contrast microscope (containing 150 to 200 cells).

### Isolation of a Triton-Insoluble Biochemical Fraction

Homogenates of cell cultures were recovered in a buffer containing 5 mM Tris-Cl, 1% Triton X-100 and a mixture of protease inhibitors (Complete, Roche) to separate the detergent-insoluble fraction (i.e., enriched in postsynaptic densities and lipid rafts) after centrifugation at 100,000 ×*g* for 1 h. The pellet was resuspended in 50 mM Hepes pH 7.4.

### Isolation of a Crude Membrane Fraction (P2) from Wild Type and Knockout Mice

Tissue homogenates from the cerebellum and forebrain were used to obtain a crude membrane fraction (P2) by differential centrifugation steps as reported (Wyneken et al., 2001).

### Co-immunoprecipitation

Cultured cell lysates (300 μg of protein) were solubilized during 2 h in solubilization buffer (50 mM Tris-HCl pH 7.5, 1% sodium deoxycholate plus proteases inhibitors), under constant agitation at 4°C. After centrifugation, the supernatant was incubated overnight with the TrkB primary antibody (Upstate # 07-225) or normal rabbit IgG (Santa Cruz). Sepharose-protein G beads (Amersham) blocked with 0.2% BSA were incubated with the protein solution for 1 h at 4°C in rotation. Then, the samples were washed five times with deoxycholate buffer and re-suspended in loading buffer. Samples were further analyzed by Western Blot.

### Western Blots

For Western Blots, protein concentration was adjusted to a final concentration of 1 mg/ml in gel-loading buffer. Proteins were separated by sodium dodecyl sulfate polyacrylamide gel electrophoresis on 10% gels and transferred to nitrocellulose membranes. The membranes were blocked for 1.5 h in 5% milk powder, incubated overnight with primary antibodies, and immunoreactivity was visualized using the ECL detection system (Amersham Buchler). For validation of eNOS antibodies in Western Blots, brain tissue of the following mouse strains were used: C57BL/6 (wild type); eNOS KO B6.129P2-Nos3tm1UNC and nNOS KO B6.129S4-Nos1tm1Plh (Jackson, Bar Harbor, ME, United States).

### Data Analysis

Average values are expressed as means ± SEM. Statistical significance of results was assessed using two-tailed Student’s *t*-test or one-way ANOVA followed by Bonferroni post-tests, as indicated.

## Results

To detect the distribution of eNOS in neuronal cell cultures, we tested the specificity of several eNOS antibodies in brain membranes of eNOS knockout mice. Clean and highly reproducible results were obtained with the monoclonal eNOS antibody of Transduction Laboratories, while the phospho-eNOS and nNOS antibodies were also proven to be reliable in Western Blots (**Supplementary Figure S1**). This eNOS antibody had already been used and reported for its specificity in diverse cells and tissues (Aschner et al., 1999; Lacza et al., 2003; Brosnihan et al., 2008). In turn, the specificity of this antibody in immunostainings was verified by knocking down the expression of eNOS usingspecific shRNA-RFP plasmids (**Supplementary Figure S2**). The shRNA1 sequence was selected for controlling the antibody specificity because the shRNA2 sequence knocked down eNOS with less efficiency (Fold change (2 ΔCt) were the following for both: shRNA1 = 0.48 ± 0.02 (*n* = 3) *vs.* shRNA2 = 0.57 (*n* = 2, not shown). In RFP-positive neurons transduced with shRNA1, no eNOS staining could be detected at day 12 *in vitro* (upper panels), while RFP-positive neurons transfected with the control shRNA revealed a punctate staining pattern (lower panels). This scenario was observed in three different neuronal cultures, in which the transduced neurons (i.e., expressing RFP, about 40% in a culture dish) were negative for eNOS. The same results were obtained with the polyclonal eNOS antibody used in **Supplementary Figure S3** (not shown). However, the polyclonal antibody did not detect any protein band in Western Blots in our hands and for this reason, the central part of the paper was performed with the monoclonal antibody.

**FIGURE 1:**
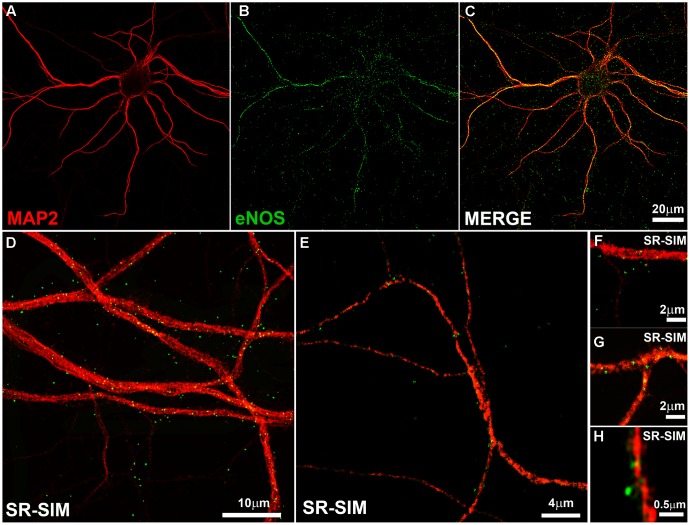
The endothelial isoform (eNOS) puncta decorate the dendritic tree. **(A–C)** Confocal microscopy shows the dendritic marker MAP2 (red) and a punctate eNOS pattern (green) in cortical cells. **(D–E)** SR-SIM microscopy in hippocampal cells using the same antibodies. **(F–H)** SR-SIM images of dendrite segments of hippocampal cells at higher magnification. The calibration bars in the corresponding panels are indicated.

We thus stained hippocampal neurons in culture with MAP2 antibody as somatodendritic marker and with the monoclonal eNOS antibody (**Figure 1**). A punctate pattern of eNOS was observed both in confocal as well as SR-SIM images at proximal and distal dendrites. With increasing magnification, dendritic spines emerging from the dendritic tree, that is densely decorated with eNOS-positive puncta, could be clearly detected (**Figure 1H**). The punctate pattern that is not associated to dendrites in the image are most likely associated to dendrites that appear in a different focal plane (**Figure 1D**). In addition, eNOS may be associated to intracellular membranes in different cell types, as the cultures contain a minor proportion of glial cells, known to express eNOS (Li et al., 2014; Munoz et al., 2015). In turn, the presence of eNOS in axons (as it is suggestive in **Figure 1E**) cannot be discarded and both may account for the positive signals that appear not clearly associated to dendrites.

**FIGURE 2:**
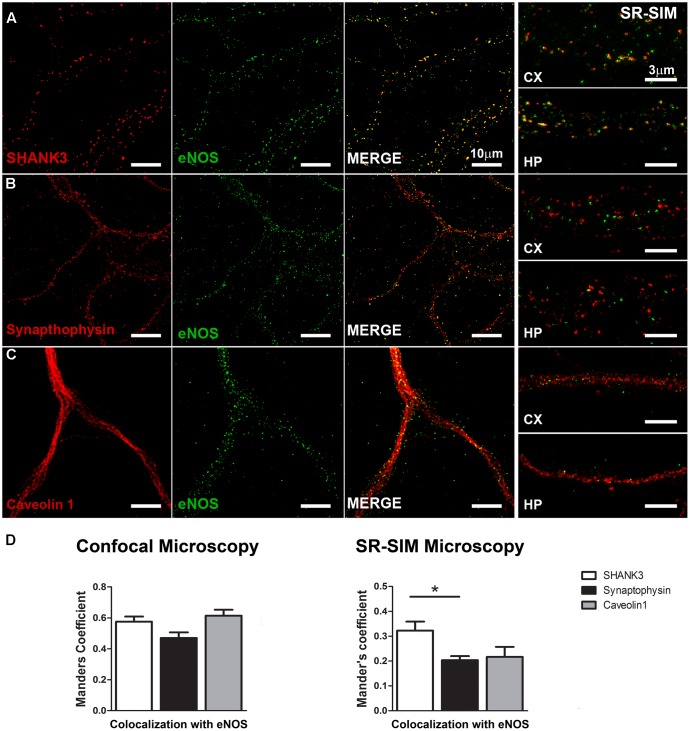
eNOS co-distributes with the postsynaptic scaffolding protein SHANK3. Confocal microscopy (left panels) and SR-SIM microscopy (right panels) of eNOS (green) with SHANK 3 (red) **(A)**, synaptophysin (red) **(B)** or caveolin 1 (red) **(C)** in hippocampal neurons (left panels) or in cortical neurons (right). **(D)** Left panel shows Mander’s coefficient calculated with confocal images while in the right panel, the same is shown with super-resolution images. *N* = 3 independent cortical culture dates, N = 4 independent hippocampal culture dates; 6 to 8 neurons per culture day (^∗^*p* < 0.05 in Student’s *t*-test when comparing SHANK3 with synaptophysin or caveolin 1 obtained from a total of 7 independent cultures).

To better explore a possible synaptic locus of expression for eNOS, we analyzed the degree of overlap between the post-synaptic protein SHANK3/ProSAP2, the pre-synaptic protein synaptophysin or the lipid raft protein caveolin 1, with eNOS along the dendritic arborization of hippocampal neurons (**Figure 2**). In eNOS and SHANK3 stainings, a significant co-localization calculated by Mander’s coefficient in confocal microscopy images (0.58 ± 0.03) was observed (**Figure 2A**). However, given that the size of dendritic spines is near the resolution limit of confocal microscopy (i.e., ≥200 nm), we also used super-resolution images (SR-SIM). In this case, the correlation coefficient decreased as expected to 0.32 ± 0.03 (**Figure 2D**). Additionally, the co-distribution coefficients of eNOS with the synaptic scaffolding protein PSD-95 confirmed the presence of eNOS in dendritic spines with a Mander’s coefficient of 0.66 ± 0.03 in confocal images and 0.3 ± 0.008 in SR-SIM images, respectively (**Supplementary Figure S3**). These results suggest that eNOS is localized in close proximity to the scaffolding proteins SHANK3 and PSD-95, major components of the postsynaptic density in dendritic spines and thus used as spine markers (Schultze et al., 2001; Grabrucker et al., 2011). In contrast, the co-distribution of eNOS with the pre-synaptic marker synaptophysin decreased significantly compared to SHANK3 when assessed by super-resolution, thus supporting a preferential post-synaptic localization of eNOS (**Figures 2B,D**). To assess whether eNOS is associated with lipid rafts, the co-distribution of the enzyme with caveolin 1 was studied (**Figures 2C,D**). In confocal microscopy and SR-SIM images, the respective Mander’s coefficients were of 0.61 ± 0.04 and 0.22 ± 0.04, respectively, supporting a partial association of eNOS with lipid rafts. Co-distribution studies with the raft marker Thy-1, showed similar results with a Mander’s coefficient of 0.65 ± 0.03 and 0.29 ± 0.01 in confocal and SR-SIM images (**Supplementary Figure S3**). These results suggest that a proportion of eNOS might be associated with raft membranes. In both cortical and hippocampal cultures, similar results for each of the postsynaptic, presynaptic and raft markers were obtained, indicating that in both culture types, eNOS is associated to dendritic spines.

**FIGURE 3:**
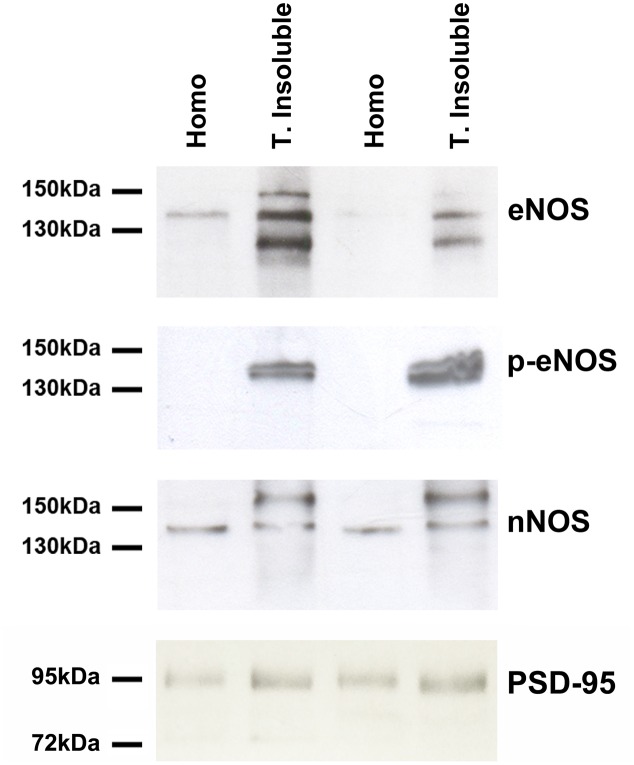
eNOS (BD Transduction) and phosphorylated eNOS (serine 1177) (BD Transduction) are present in triton-insoluble biochemical preparations. Representative Western Blots of cell homogenates (Homo) and detergent-insoluble fraction (T. insoluble, i.e., enriched in post-synaptic densities and lipid rafts) of primary cell cultures. Equal amounts of protein were loaded per lane.

To further confirm our previous results, we prepared extracts of the Triton-X insoluble fraction from primary cultures, known to be enriched in postsynaptic densities (PSDs) as well as lipid rafts. Western Blots with antibodies against eNOS, eNOS phosphorylated on serine 1177 as well as nNOS were performed (**Figure 3**). eNOS and p-eNOS were enriched in the Triton-insoluble fraction of both cortical and hippocampal cultured cells (left and right pair of lanes, respectively. In contrast, nNOS was not enriched in the Triton-insoluble fraction when compared to the total homogenate. As expected, the scaffolding protein PSD-95 indicated a relative enrichment in the Triton-insoluble fraction over homogenates.

**FIGURE 4:**
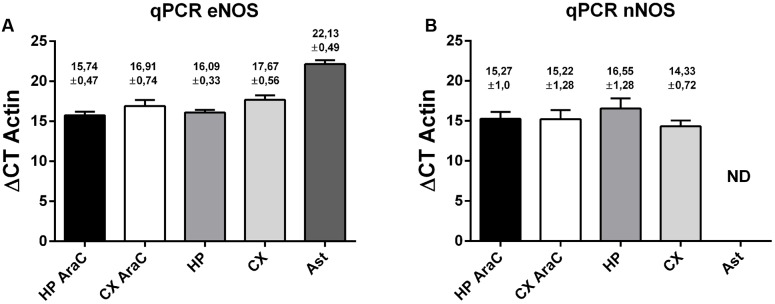
Expression profile of eNOS and nNOS in neuronal cells. eNOS mRNA expression **(A)** and nNOS mRNA expression **(B)** measured by quantitative PCR of the following cell cultures: neurons in the presence of AraC to reduce glia proliferation in cultures derived from hippocampus (HP AraC) or from the cerebral cortex (CX AraC); neurons in mixed neuron/glia cultures derived from the hippocampus (HP) or cerebral cortex (CX); or pure astrocyte cultures (Ast). Bar graph showing the mean ± SEM in ΔCt values normalized against geometric mean of actin and GAPDH as reference gene. Mean Ct ± SEM values are indicated on each bar (data obtained from *n* = 5 independent hippocampal cultures and *n* = 6 independent cortical cultures).

Then, quantitative PCR was performed to detect eNOS (**Figure 4**, left panel) and nNOS (**Figure 4**, right panel) transcripts in cell cultures. Using specific primers (see also **Supplementary Figure S2**), we could detect similar amounts of eNOS in cortical and hippocampal neurons grown virtually without glia (using AraC) and in mixed neuronal cultures, suggesting that astrocytes are not major contributors to these measurements. eNOS was also present in astrocytes, although at lower levels. In turn, nNOS could also be detected in neurons in the same cultures, although in astrocytes cycle thresholds were over 35, and thus considered a negative reaction in our experimental conditions.

**FIGURE 5:**
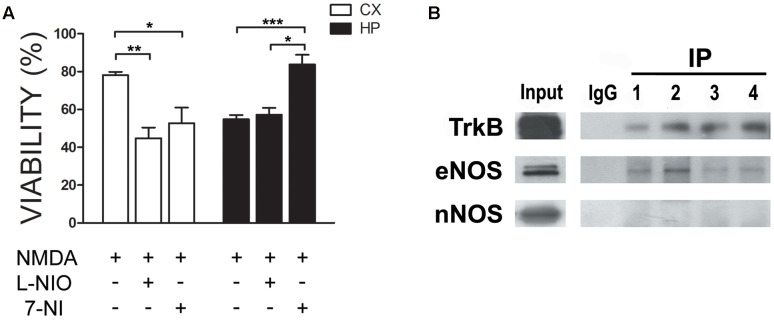
eNOS protects cortical neurons from excitotoxicity. **(A)** Neurons were incubated with 30 μM NMDA (+10 μM CNQX, 2 μM Nimodipine, 1 μM TTX) in the presence or absence of 1 μM of 7-Nitroindazole (7-NI), a preferential nNOS inhibitor or 10 μM of N5-(1-Iminoethyl)-L-ornithine (LNIO), a preferential eNOS inhibitor. Cell viability was measured by the Trypan Blue exclusion test (^∗^*p* < 0.05; ^∗∗^*p* < 0.01; ^∗∗^*p* < 0.001). HP = hippocampal cultures; CX = cortical cultures. **(B)** TrkB was immunoprecipitated from cell culture homogenates to detect eNOS in the immunoprecipitates. Western Blots of the samples were loaded as follows: input, negative control (rabbit IgG) and TrkB immunoprecipitates from hippocampal cultures (lanes 1 and 2) or cortical cultures (lanes 3 and 4).

In previous work, we had shown that nitric oxide (NO) is produced in response to the neurotrophin BDNF in neuronal cultures. Moreover, this NO resulted in neuroprotection (Sandoval et al., 2011). We thus as a first insight into a putative functional role of eNOS, we performed an excitotoxicity assay stimulating cultures with NMDA in the presence or absence of 1 μM of 7-Nitroindazole (7-NI), a preferential nNOS inhibitor or 10 μM of N5-(1-Iminoethyl)-L-ornithine (LNIO), a preferential eNOS inhibitor (El-Mas and Abdel-Rahman, 2013; Li et al., 2014) (**Figure 5A**). As we had shown previously, both culture types are selectively sensitive to this NMDA concentration: while hippocampal viability decreased, no effect was observed in cortical cultures. However, when eNOS was inhibited with LNIO, cortical viability decreased, indicating a protective effect of this NO source. In contrast, no further decrease of viability was observed in hippocampal cultures. In turn, 7-NI, preferentially inhibiting nNOS, a NO source that is deleterious under excitotoxic conditions in hippocampal neurons, protected neurons from cell death. These results confirm a differential response of both culture types to NMDA and that in cortical cells, NO is protective while in hippocampal cells, nNOS-derived NO is deleterious. Based on previous data, we hypothesized that eNOS, having a neuroprotective function, could be functionally be coupled to the BDNF receptor TrkB. Thus, we performed immunoprecipitations of TrkB (**Figure 5B**) and eNOS. While TrkB could be easily immunoprecipitated and eNOS, but not nNOS, detected in the precipitates, we were not able to immunoprecipitates eNOS. This is probably related to the nature of the antibody, because different co-authors of the paper using different solubilization buffers had the same negative results. Thus, these results suggest that eNOS might be a source of protective NO in neurons.

## Discussion

In this study, we confirmed the presence of eNOS in primary cultures of neurons. Furthermore, we show punctate eNOS staining and co-distribution with synaptic scaffolding proteins, compatible with its localization within dendritic spines. Although, previous research had found a widespread distribution of eNOS in the cell body and dendrites of hippocampal pyramidal cells (O’Dell et al., 1994), it remained undetermined whether it is preferentially localized at synaptic or extra-synaptic sites. Thus, our finding of a postsynaptic localization in central neurons suggests that its contribution to the regulation of neuronal function might have been underestimated. The importance of this finding is that it adds further support to the increasing evidence for eNOS playing an important role in physiological or pathophysiological processes in the brain. As a first insight into a functional role of eNOS, our results further suggest a neuroprotective role, particularly evident under excitotoxic conditions.

### eNOS vs. nNOS in Neurons and in CNS Function

The specificity of the monoclonal eNOS antibody used by us has been tested elsewhere both in endothelial tissue as well as in the brain (Lacza et al., 2003; Steinert et al., 2008). The most widely accepted view is that the contribution of eNOS-derived NO to neuronal function is from endothelium diffusion and spillover (Steinert et al., 2010). In the medial nucleus of the trapezoid body, a structure of the auditory brainstem, nNOS but not eNOS is located at postsynaptic sites (Steinert et al., 2008). Although in nNOS KO mice the regulation of postsynaptic excitability was shown to be abolished in the medial nucleus of the trapezoid body, this was not reported for CA3 pyramidal neurons, thus leaving unresolved which isoform is responsible for this effect (Steinert et al., 2011). In cultured pyramidal neurons of hippocampal and cortical origin (Rameau et al., 2004, 2007), nNOS has also been detected thus constituting a potential source of NO in primary cultures. However, in these studies, the specificity of the antibody with respect to the eNOS isoform was not addressed.

In the brain, eNOS is expressed by several neuronal types such as olfactory sensory neurons, where it might influence behavioral changes (Brunert et al., 2009; Sung et al., 2014) or in dorsal root ganglia neurons were it modulates inflammatory pain (Borsani et al., 2013). In addition, several neuronal types of the chicken retina contain eNOS and produce nitric oxide (Tekmen-Clark and Gleason, 2013). In turn, it is not clear which NOS isoform contributes to BDNF/TrkB-dependent NO synthesis in cortical neurons (Sandoval et al., 2011; Kolarow et al., 2014), a process that might be involved in the regulation of LTP. eNOS is consistently expressed in neuronal populations under different pathological conditions, and it has been proposed that an enhanced neuronal expression exerts neuroprotective actions (De Palma et al., 2008). We do not know whether the standard cell culture conditions used in this study may represent a condition that triggers neuronal eNOS expression. In that line, eNOS expression in astrocytes is induced under pro-inflammatory conditions *in vivo* (Iwase et al., 2000).

While there is no doubt regarding the participation of eNOS in the establishment of LTP (O’Dell et al., 1994; Hopper and Garthwaite, 2006), its contribution to NO production has been claimed to be either of endothelial (Kantor et al., 1996; Hopper and Garthwaite, 2006) or neuronal (Haley et al., 1996) origin. Thus, we decided to further explore its role in excitotoxicity (Sandoval et al., 2011). As expected, we could confirm that eNOS-derived NO is protective in cortical neurons because its pharmacological inhibition abolished the relative resistance of cortical neurons to 30 μM NMDA. In hippocampal neurons, that already were vulnerable to this NMDA concentration, no further effect was observed suggesting that both proteins form part of a common signaling pathway. Interestingly, eNOS co-immunoprecipitated with TrkB in both culture types, suggesting that eNOS-dependent NO production could be associated to BDNF. In that line, the role of NO in neurotransmission and its regulation by BDNF needs to be addressed in the future.

### Synaptic Targeting of eNOS

The expression of the eNOS isoform in neurons has been addressed in a few studies studying brain tissue, but never in neuronal cultures, in which many cell biology experiments are performed. Consistent with our results, eNOS has been detected by mass spectrometry in highly purified postsynaptic densities obtained from the mouse hippocampus in 3 biological replicates (Distler et al., 2014). The higher co-distribution of eNOS with scaffolding proteins such as PSD-95 located at less than 50 nm from the plasma membrane, or with SHANK3, located at over 50 nm from the plasma membrane when compared with synaptophysin, confirms its preferential presence in dendritic spines over pre-synaptic boutons (Dosemeci et al., 2016). NOS isoforms can undergo tissue-specific regulation and targeting by protein-protein interactions. In endothelial cells, eNOS is mainly targeted to the plasma membrane or intracellularly, to the Golgi apparatus. The clustered staining pattern of eNOS in cultured neurons is compatible with its membrane targeting and with an interaction with caveolin-1 in lipid rafts (Head et al., 2014). Furthermore, it does colocalize with Thy-1, a specific marker of non-caveolar lipid rafts (Yao et al., 2009). The exact meaning of this finding is still unknown, but it suggests that in addition to interactions with caveolin-1, e-NOS is targeted by additional protein-protein interactions to other cellular domains. In keeping with that observation, eNOS interacts with and is activated by dynamin-2, a large GTPase involved in vesicular budding and internalization of caveolae as well as membrane trafficking events (Cao et al., 2001). Interestingly, dynamin-2 is an isoform-specific binding partner of the Shank family of proteins (Okamoto et al., 2001). Thus, a molecular link between eNOS and Shank isoforms can be possibly mediated by dynamin-2 (Kondrikov et al., 2010). Furthermore, eNOS in spines could be anchored to the actin cytoskeleton, characterized by a dynamic and exquisite regulation (Frotscher et al., 2014; Spence and Soderling, 2015). However, a fine spatial resolution and identification of protein partners has to be addressed with additional methods such as electron microscopy and co-immunoprecipitation. Although in TrkB precipitates, eNOS could be detected, we could not immunoprecipitate eNOS. This has been confirmed in several experimental designs and thus, the antibodies in use seem not to be suitable for immunoprecipitation. Alternative methods should been employed in the future to assess the presence of eNOS in protein complexes, such as expression of tagged eNOS in neurons that would allow affinity isolation. It cannot be excluded that a large proportion of eNOS in neurons might also be associated with intracellular membranes, such as the Golgi apparatus, an issue that was not investigated in the present study.

Taken together, our results strongly support an association of eNOS with excitatory synapses, suggesting a functional contribution to synaptic function.

## Author Contributions

Conceived and designed the experiments: UW, AM, MV-G. Performed the experiments: AC, AM, SS. JB-A performed experiments (immunoprecipitations). Contributed reagents/materials/analysis tools: FN and TK. Analyzed the data: AC, MV-G. Wrote the paper: UW, CL, JF.

## Conflict of Interest Statement

The authors declare that the research was conducted in the absence of any commercial or financial relationships that could be construed as a potential conflict of interest.

## Supplementary Material

FIGURE S1 eNOS content in membranes of different tissues of eNOS and nNOS knock out mice reveal the specificity of the eNOS antibody used in this study. The same blots were reprobed with the nNOS and phospho-eNOS antibodies. Representative Western Blots after loading 20 μg of protein/per lane except in lane 1, in which 12.5% was loaded (∼2.5 μg). Lane 1 = positive eNOS control (sheep brain homogenate); lane 2 (KO1e) = eNOS KO mice, cerebellum homogenates; lane 3: WT = wild type mice, crude membrane fraction of brains; lane 4 (A): rat aorta homogenates of wild type mice; lane 5 (KOn) = nNOS KO mice, crude membrane fraction of brains; lane 6 (KO1e) = eNOS KO mice, crude membrane fraction of brains.

FIGURE S2 Knockdown of eNOS with lentiviral shRNA show the specificity of the eNOS antibody used in this study. **(A)** Epifluorescence microscopy of eNOS (right panels, green) and transduced cells (left panels, red) in hippocampal neurons. **(B)** eNOS mRNA expression (left panel) and nNOS mRNA expression (right panel) measured by quantitative PCR of hippocampal neurons transduced with a lentivirus encoding a scrambled shRNA (control) or a shRNA against eNOS. Bar graph showing the mean ± SEM fold change normalized against actin as reference gene (data obtained from *n* = 3 independent hippocampal cultures) (^∗^*p* < 0.05 in one-way ANOVA followed by Bonferroni).

FIGURE S3 eNOS co-distributes with the postsynaptic scaffolding protein PSD-95 and with the raft marker Thy-1. Confocal microscopy (left panels) and SR-SIM microscopy (right panels) of eNOS (green) and Thy-1 (red) **(A)** or PSD-95 (red) **(B)** in hippocampal neurons (left panels) or in hippocampal and cortical neurons (right). **(C)**: Left panels show Mander’s coefficient calculated with confocal images while in the right panels, the same is shown with SR-SIM images. For comparison of co-distribution coefficients with SHANK3, the same data of **Figure 2** was used. *n* = 7 independent cell culture dates and 6 to 8 neurons per culture day.
