# Supplementary figures and images for: Endothelial Nitric Oxide Synthase Is Present in Dendritic Spines of Neurons in Primary Cultures
Source: Front Cell Neurosci. 2017 Jul 4;11:180. doi: 10.3389/fncel.2017.00180 (PMC5495831; doi:10.3389/fncel.2017.00180)

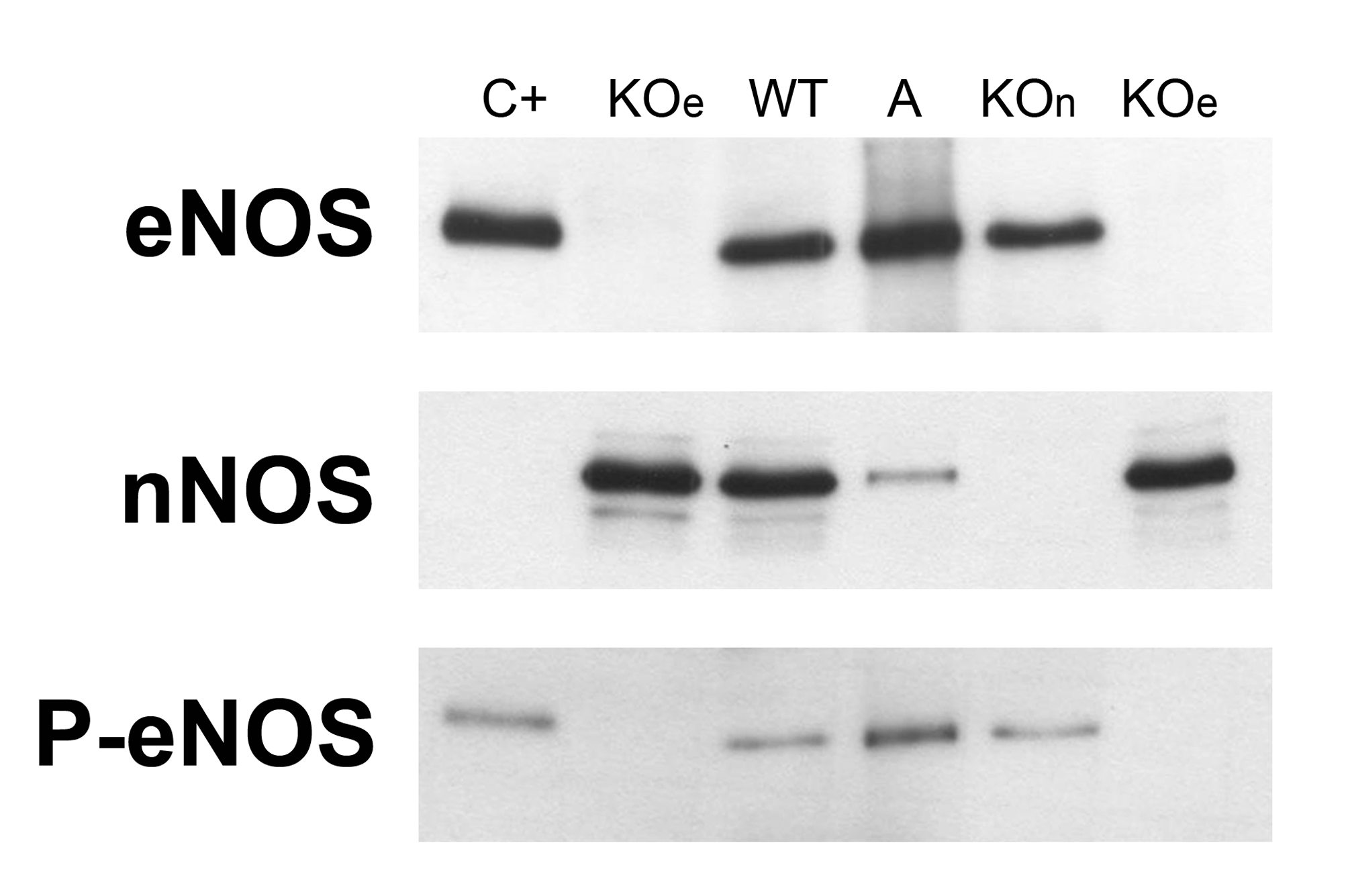

Supplement: FIGURE S1 — eNOS content in membranes of different tissues of eNOS and nNOS knock out mice reveal the specificity of the eNOS antibody used in this study. The same blots were reprobed with the nNOS and phospho-eNOS antibodies. Representative Western Blots after loading 20 μg of protein/per lane except in lane 1, in which 12.5% was loaded (∼2.5 μg). Lane 1 = positive eNOS control (sheep brain homogenate); lane 2 (KO1e) = eNOS KO mice, cerebellum homogenates; lane 3: WT = wild type mice, crude membrane fraction of brains; lane 4 (A): rat aorta homogenates of wild type mice; lane 5 (KOn) = nNOS KO mice, crude membrane fraction of brains; lane 6 (KO1e) = eNOS KO mice, crude membrane fraction of brains. [file Image_1.TIF]

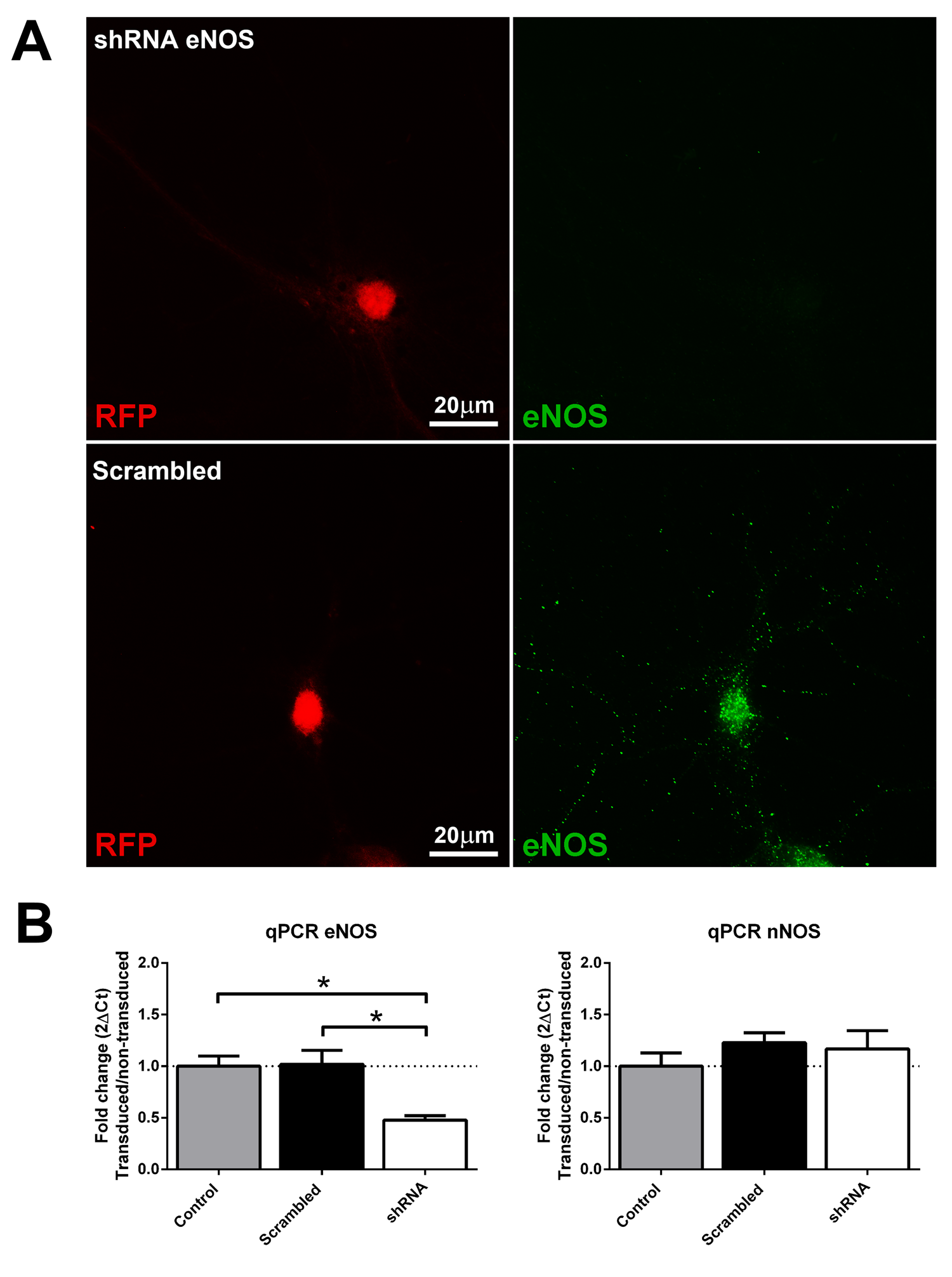

Supplement: FIGURE S2 — Knockdown of eNOS with lentiviral shRNA show the specificity of the eNOS antibody used in this study. (A) Epifluorescence microscopy of eNOS (right panels, green) and transduced cells (left panels, red) in hippocampal neurons. (B) eNOS mRNA expression (left panel) and nNOS mRNA expression (right panel) measured by quantitative PCR of hippocampal neurons transduced with a lentivirus encoding a scrambled shRNA (control) or a shRNA against eNOS. Bar graph showing the mean ± SEM fold change normalized against actin as reference gene (data obtained from n = 3 independent hippocampal cultures) (∗p < 0.05 in one-way ANOVA followed by Bonferroni). [file Image_2.TIF]

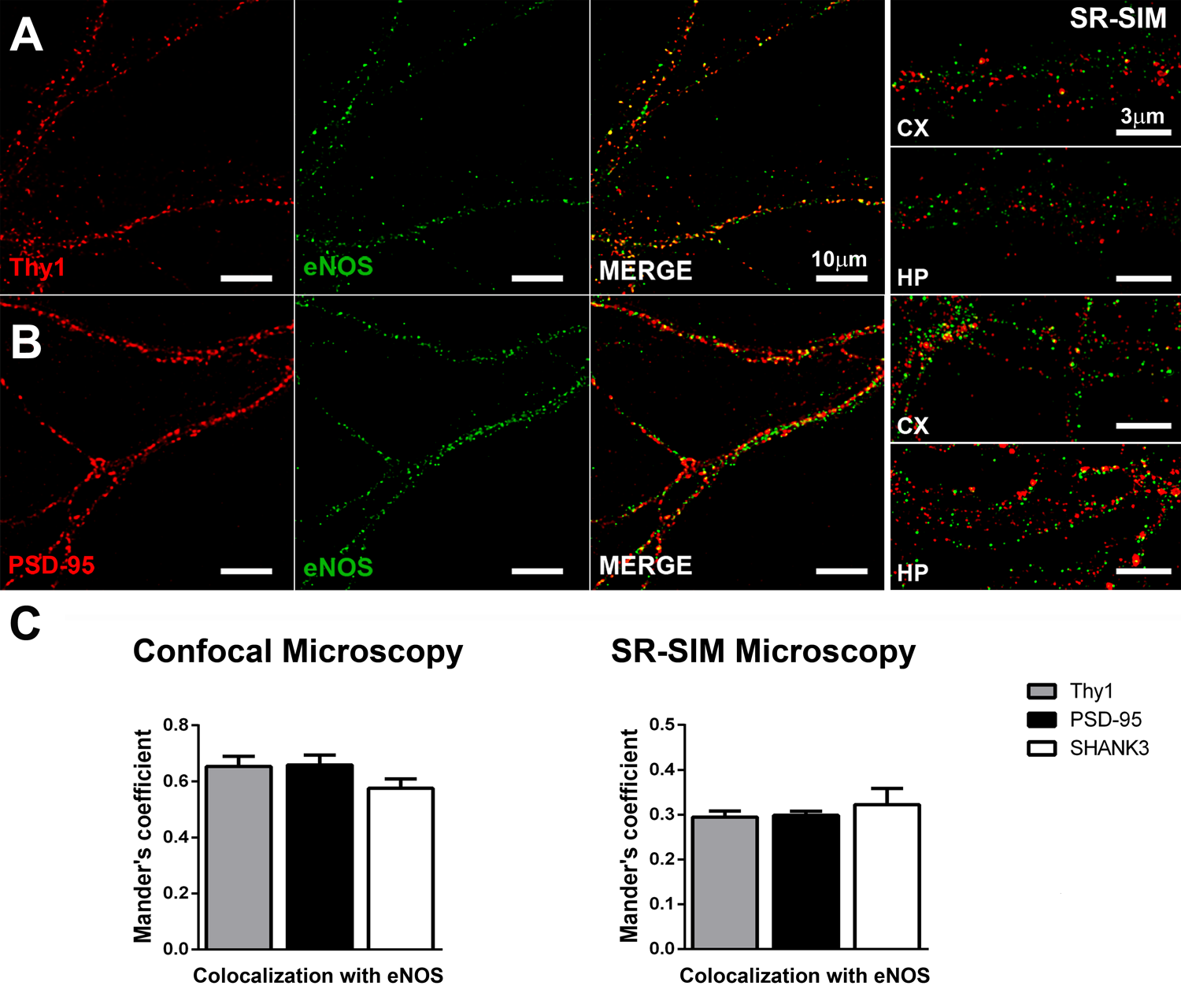

Supplement: FIGURE S3 — eNOS co-distributes with the postsynaptic scaffolding protein PSD-95 and with the raft marker Thy-1. Confocal microscopy (left panels) and SR-SIM microscopy (right panels) of eNOS (green) and Thy-1 (red) (A) or PSD-95 (red) (B) in hippocampal neurons (left panels) or in hippocampal and cortical neurons (right). (C): Left panels show Mander’s coefficient calculated with confocal images while in the right panels, the same is shown with SR-SIM images. For comparison of co-distribution coefficients with SHANK3, the same data of Figure 2 was used. n = 7 independent cell culture dates and 6 to 8 neurons per culture day. [file Image_3.TIF]
